# Magnetic, Fluorescence and Transition Metal Ion Response Properties of 2,6-Diaminopyridine Modified Silica-Coated Fe_3_O_4_ Nanoparticles

**DOI:** 10.3390/molecules21081066

**Published:** 2016-08-15

**Authors:** Yunhui Zhai, Ruijuan Song, Changhu Zhang, Qun He, Quan Han, Yingjuan Qu

**Affiliations:** 1 Department of Chemistry and Chemical Engineering, Xi’an University, Xi’an 710065, China; song_ruijuan66@126.com (R.S.); zhangchanghu12@163.com (C.Z.); xahquan@hotmail.com (Q.H.); qu_yingjuan66@126.com (Y.Q.); 2 Department of Chemistry and Chemical Engineering, Lanzhou University, Lanzhou 730000, China; he_qun66@126.com

**Keywords:** multi-functional nanoparticles, magnetic, fluorescence, metal ions response

## Abstract

Multi-functional nanoparticles possessing magnetic, fluorescence and transition metal ion response properties were prepared and characterized. The particles have a core/shell structure that consists of silica-coated magnetic Fe_3_O_4_ and 2,6-diaminopyridine anchored on the silica surface via organic linker molecules. The resultant nanoparticles were found by transmission electron microscopy to be well-dispersed spherical particles with an average diameter of 10–12 nm. X-ray diffraction analysis suggested the existence of Fe_3_O_4_ and silica in/on the particle. Fourier transform infrared spectra revealed that 2,6-diaminopyridine molecules were successfully covalently bonded to the surface of magnetic composite nanoparticles. The prepared particles possessed an emission peak at 364 nm with an excitation wavelength of 307 nm and have a strong reversible response property for some transition metal ions such as Cu^2+^ and Zn^2+^. This new material holds considerable promise in selective magneto separation and optical determination applications.

## 1. Introduction

Since the surprising special performance characteristics of nanometer materials were discovered, many nanometer-sized materials have been fabricated by scientists and engineers. Their properties in mechanics, electromagnetism, optics and other physical and chemical characters have been investigated in detail [1,2,3]. Currently, nanometer scale composites having multifunctional properties are attracting tremendous interest, largely due to their unique coupled behaviors. For instance, materials with both photo-luminescent and magnetic properties could be used in a wide range of applications in biological systems, selective separations and chemical determinations including serving as luminescent markers as well as magnets controlled by an external field [4,5,6,7,8,9,10,11].

Luminescent nanomaterials involve dots, metal nanoparticles (gold and silver) and dye-doped polymer or silica nanoparticles. Among them, silica-coated and organic-dye-doped iron oxide nanoparticles have shown great promise as biomarkers due to their advantages such as low toxicity, biocompatibility and chemical stability [12,13]. Using tris(2,2′-bipyridine) ruthenium(II) chloride (Rubpy), Simard et al. prepared a kind of reagent-doped silica shell on the surface of iron oxide nanoparticles [14]. Ren et.al prepared rhodamine B doped silica Fe_3_O_4_ magnetic nanoparticles via the lay by lay method [15]. These nanomaterials possessed good magnetic and fluorescent properties simultaneously. However, they must be further modified by other function groups or reagents if they are used in selective recognition, as chemical markers or for analyte determination. In addition, the fluorescent reagent leakage problem needed to be resolved since they were physically doped into the silica net during preparation [16]. An effective method has been developed recently in which the reagents, which could react with the target and give response signals, were coupled to silane-coupling agent by isothiocyanate or carboxylic acid presented in the molecules and then subsequently co-hydrolyzed in the presence of tetraethoxysilane (TEOS) during the formation of the silica shell coating of the magnetic cores [17,18,19]. Moreover, these functional silanization reagents were also applied to the preparation of surface modifying silica-coated magnetic nanoparticles for use in fluorescent response time delay because the dye or reagent is trapped in the silica shell of such composite materials [20,21,22,23]. All these materials exhibited good magnetic and selective fluorescence response properties for the target ions. However, synthesis and purification of the functional silanization reagent was a necessary step before co-hydrolyzing with TEOS or modifying on the surface of magnetic silica for these two methods.

**Figure 1:**
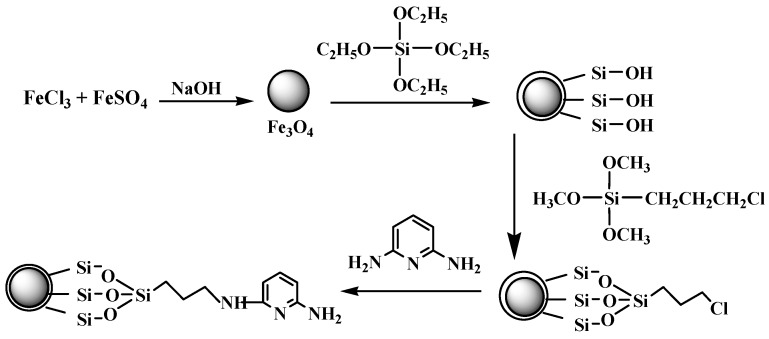
Scheme illustration of the preparation of 2,6-Diaminopyridine (DAPD)-SMN.

It was, therefore, thought worthwhile to develop a method to prepare multi-functional nanoparticles possessing magnetic, fluorescence and metal ion response properties more easily and efficiently. Zou and coworkers have synthesized super paramagnetic silica-coated Fe_3_O_4_ nanocrystals by chemical co-precipitation and hydrolysis of tetraethyl orthosilicate [24]. The particles have good dispersion with the carboxyl groups binding on the surface through reaction of –NH_2_ and glutaric anhydride. Their method was simple and efficient. Very recently, Sadeghia et al. synthesized quercetin-modified silica-coated magnetic Fe_3_O_4_ nanoparticles by using 3-aminopropyltriethoxysiliane as the coupling agent [25]. So this paper extended their method to prepare a new kind of multifunctional nanoparticles composed of a super paramagnetic core and small molecule-dye-reagent-modified silica shell. 2,6-Diaminopyridine (DAPD), a familiar and simple organic dye containing two amino groups and one pyridine ring in the molecule (Figure 1), was chosen as the modifying reagent because of its favorable coordination capacity for some metal ion and its fluorescence and metal ions response properties [26]. The obtained composite materials were characterized by transmission electron microscopy (TEM), X-ray powder diffraction (XRD), vibration sample magnetometer (VSM), Fourier transform-infrared (FT-IR) spectroscopy and fluorescence spectroscopy. The characteristics of this new material including particle size, structure, morphology, magnetization, and fluorescent response properties for some transition metal ions (Cu^2+^, Zn^2+^, Cd^2+^, Ni^2+^, Co^2+^ and Pd^2+^), are also presented in detail.

## 2. Results and Discussion

### 2.1. Size, Morphology, Crystallization and FT-IR Spectra of the As-Prepared Nanoparticles

The amorphous silica could directly coat on the magnetic nanoparticles via the hydrolysis of a sol-gel precursor TEOS [27]. The iron oxide surface has a strong affinity toward silica, so no primer is required to promote its deposition and adhesion to silica. A surface coupling agent of CPS was employed as a binder to immobilize DAPD onto the silica-coated Fe_3_O_4_ nanoparticles through reaction of –NH_2_ and –Cl.

**Figure 2:**
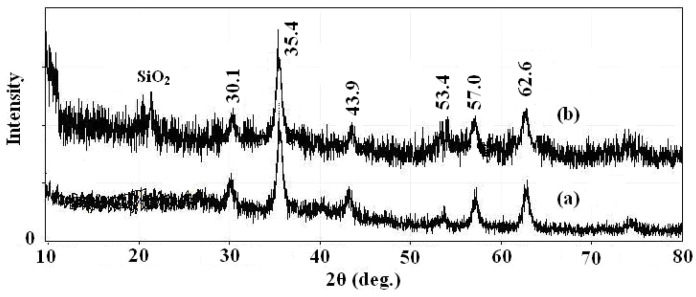
X-ray powder (XRD) patterns of (**a**) Fe_3_O_4_ nanoparticles and (**b**) SMN.

In order to fully characterize the core and shell of the synthesized magnetic nanoparticles for morphology, structure and functional agent, different techniques such as XRD, TEM and IR were used. The XRD spectrums of prepared Fe_3_O_4_ nanoparticles and SMN particles are shown in Figure 2, along with the Joint Committee on Powder Diffraction Standards (JCPDS) reference patterns of magnetite Fe_3_O_4_ (No. 19-629). A clean Fe_3_O_4_ cubic spinel phase can be confirmed. Using the most intense peak in Fe_3_O_4_ nanoparticles XRD pattern (Figure 2a), the particle sizes of approximately 7 nm was estimated by the Debye-Scherer formula [28]. The characteristic peaks of pure Fe_3_O_4_ nanoparticles at 2θ = 30.1, 35.4, 43.9, 53.4, 57.0 and 62.6 were also observed for silica-coated Fe_3_O_4_ nanoparticles (Figure 2b), which confirms the presence of the crystalline structure of the magnetite. Besides the peak of iron oxide, the XRD pattern of SMN particles presented a broad featureless XRD peak at low diffraction angle, which corresponded to the amorphous state SiO_2_ shells.

**Figure 3:**
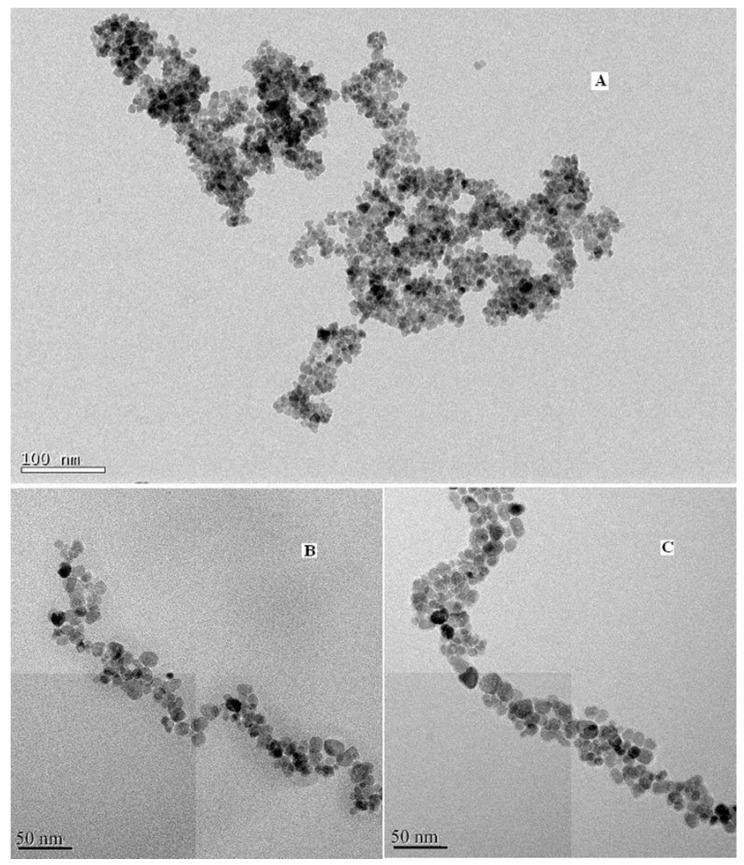
Transmission electron microscopy (TEM) images of (**A**) Fe_3_O_4_ nanoparticles; (**B**) SMN and (**C**) DAPD-SMN.

To further affirm the size and morphology of the obtained material, TEM images of the blank, coated and modified magnetic nanoparticles were taken and shown in Figure 3, respectively. It can be seen that the blank Fe_3_O_4_ particles are spherical with an average particle size of about 7–9 nm, which is consistent with the XRD results. Figure 3c illustrates that the size of DAPD-SMN is about 10–12 nm.

**Figure 4:**
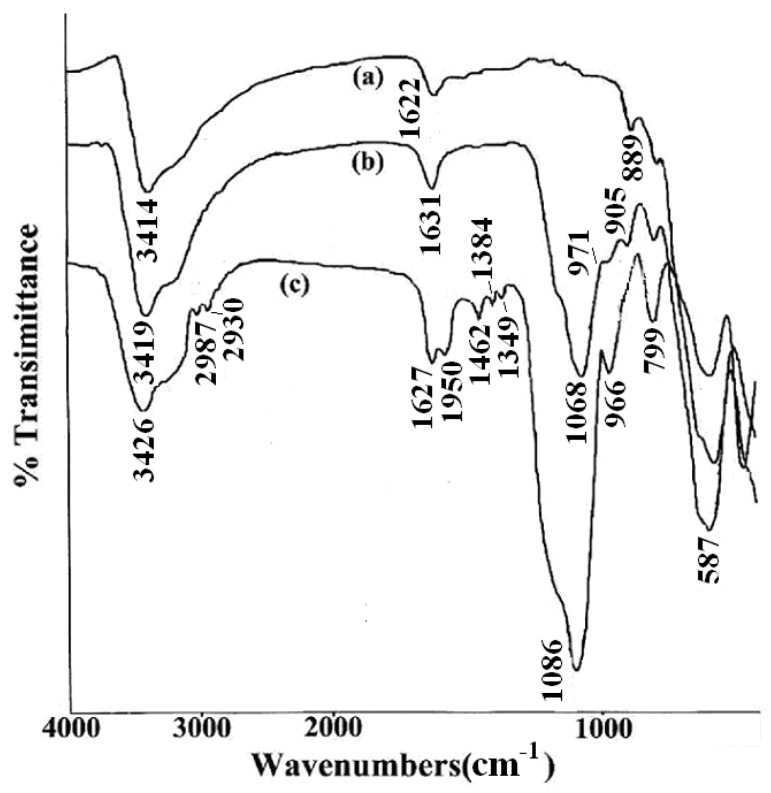
FTIR spectra of (**a**) Fe_3_O_4_ nanoparticles; (**b**) SMN and (**c**) DAPD-SMN.

To ascertain the presence of DAPD on SMN, FT-IR spectra were obtained from Fe_3_O_4_, SMN and DAPD-SMN. From the FI-IR spectra shown in Figure 4, it can be seen that the characteristic peak of Fe_3_O_4_ magnetic appeared at 587 cm^−1^. This band was shifted to high wave number compared to the Fe–O bond peak of bulk magnetite at 570 cm^−1^ due to the nanoparticle size [29]. The Si–O–Si bond’s asymmetric stretching vibration at 1068 cm^−1^ and symmetric stretching vibration at around 800 cm^−1^ appear in both SMN (Figure 4b) and DAPD-SMN (Figure 4c) spectra, which indicates that the silica has successfully coated on the surface of Fe_3_O_4_ nanoparticles by hydrolysis of TEOS. Moreover, the peaks around 1590, 1462 and 1350 cm^−1^ occurred in DAPD-SMN spectra could be assigned as features of benzene and amine in the DAPD molecules, while the presence of absorption bands around 2865 cm^−1^ and 2930 cm^−1^ corresponded to symmetrical and asymmetrical of CH_2_ stretching vibrations. The broad absorption bands at about 3420 and 1630 cm^−1^ in all the spectra mainly originate from the –OH vibrations in H_2_O. Consequently, the FT-IR spectra provided supportive evidence that DAPD attached to SMN via a reaction of –Cl and –NH_2_.

### 2.2. Magnetic Properties of the Prepared Nanoparticles

**Figure 5:**
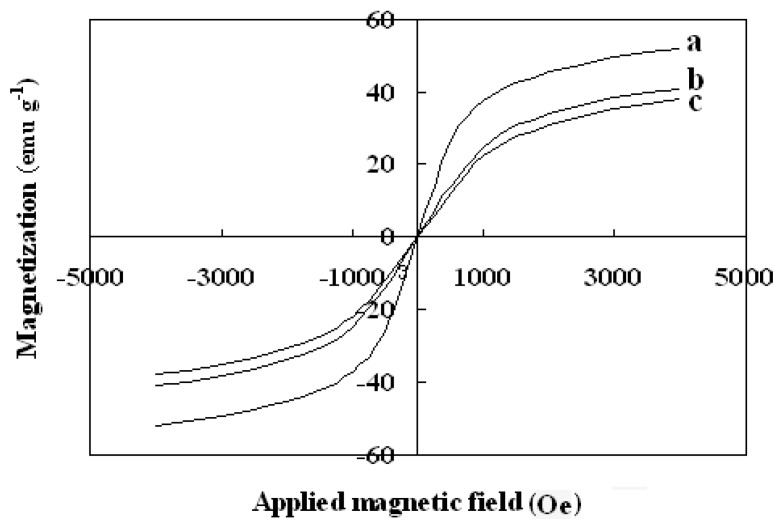
Vibration sample magnetometer (VSM) magnetization curves of (**a**) Fe_3_O_4_ nanoparticles; (**b**) SMN and (**c**) DAPD-SMN measured at 300 K.

**Figure 6:**
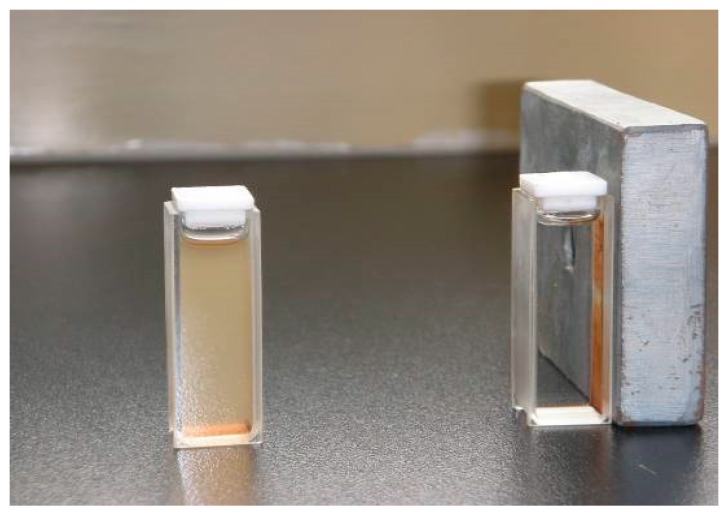
The dispersion and separation effect before and after using external magnetic field.

The magnetic properties of the synthesized nanoparticles were studied by VSM. Figure 5 shows the hysteresis loops of the Fe_3_O_4_, SMN and DAPD-SMN at room temperature. The saturation magnetization (M_s_) obtained for plain iron oxide was 52 emu·g^−1^, while this value for SMN and DAPD-SMN were about 41 emu·g^−1^ and 38 emu·g^−1^, respectively. In spite of these low magnetization values with respect to magnetization of pure Fe_3_O_4_ nanoparticles, which was owing to a decrease in the surface moments of the magnetic nanoparticles by non-magnetic silica coating, magnetic separation by a conventional magnet is still sufficient. The dispersion and separation effect before and after using an external magnetic field is shown in Figure 6.

### 2.3. Optical Properties of the Prepared Nanoparticles

**Figure 7:**
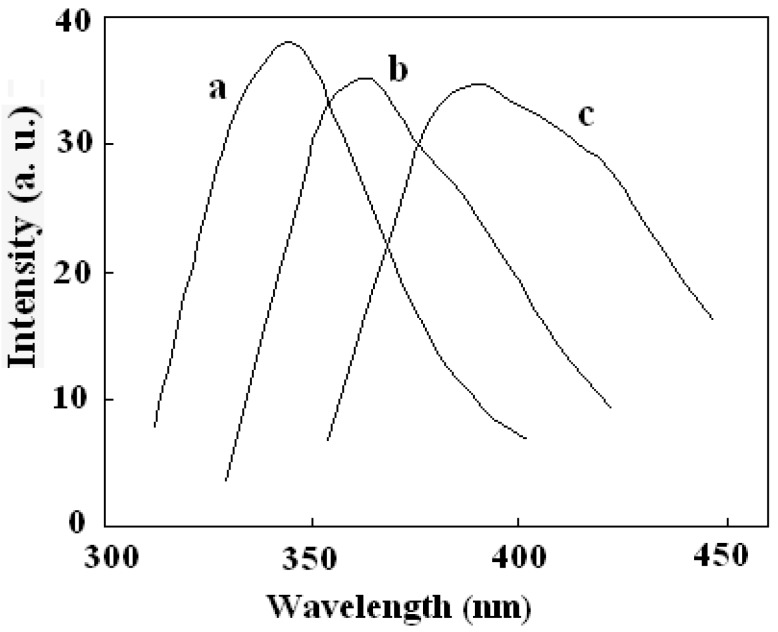
Fluorescence emission spectra of (**a**) DAPD; (**b**) DAPD-SMN and (**c**) DAPD-SMN-Cu^2+^ The excitation wavelengths are 307 nm, 310 nm and 334 nm, respectively. *C*_DAPD-SMN_ = 20 mg·L^−1^, *C*_Cu2+_ = 1 × 10^−^^5^ mol·L^−1^.

The fluorescence properties of the multifunctional nanocomposite were investigated. All tests were performed in ethanol solution. Figure 7 showed the emission spectra of DAPD, DAPD-MNP before and after adding Cu^2+^. The maximum emission of DAPD was determined to be at 346 nm with the excitation at 307 nm. However, the emission peak moved to 364 nm when the reagent combined on the surface of magnetic nanoparticles (Figure 7b). Aminopyridine is the intramolecular charge transfer fluorescent reagent which contains a pyridine ring and –NH_2_ groups. With photo excitation, intramolecular charge transfer takes place between N atoms and the pyridine ring, which results in fluorescence. When the aminopyridine was bonded to the surface of magnetic nanoparticles, the degree of the molecule’s freedom was restricted, and the –NH groups were connected with methylene on the surface of SMN particles compared with the reagent blank (see Figure 1). Therefore, a remarkable red shift was observed in its fluorescence emission.

**Figure 8:**
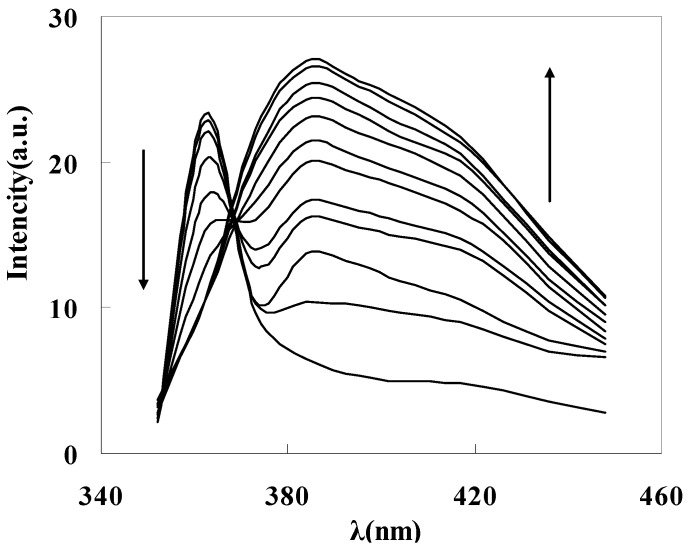
Fluorescence spectra of DAPD-SMN in ethanol at different concentration of Cu^2+^. *C*_DAPD-SMN_ = 20 mg·L^−1^, *C*_Cu2+_ = 0~3 × 10^−6^ mol·L^−1^, ***λ***_ex_ = 334 nm.

To get the binding properties of DAPD-MNP towards transition metal ions, Cu^2+^ was selected as a typical target for testing according to the Irving-Williams rule. When excess Cu^2+^ was added into the dispersed system, the fluorescence emission peak moved to 385 nm (Figure 7c), which indicates that the aminopyridine groups located on the surface of magnetic nanoparticles have effectively coordinated with Cu^2+^. The formation of coordination bonds increases the conjugation degrees, so that the emission peak has a remarkable red shift. Figure 8 is the fluorescence spectra of DAPD-MNP with gradually increasing Cu^2+^ concentration. It can be seen that the emission intensity of 364 nm gradually decreases and the 385 nm peak is increased by degrees. A plot of fluorescence intensity function with the concentration of Cu^2+^ at 385 nm was fitted. Good linear correlation was obtained at the concentration of 2.25 × 10^−7^ − 2.50 × 10^−6^ mol·L^−1^. The linear equation is y = 5.0306x + 15.245 with the correlation coefficient R^2^ = 0.9975.

**Figure 9:**
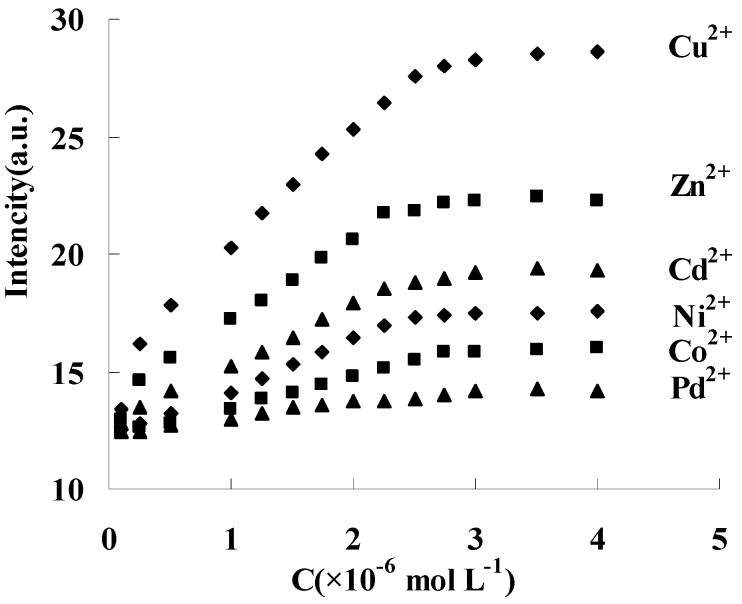
Effect of transition metal ions upon the fluorescence intensity of DAPD-SMP in ethanol at 385 nm, *C*_DAPD-SMN_ = 20 mg·L^−1^, ***λ***_ex_ = 334 nm.

Similar fluorescence responses were also obtained between DAPD-MNP and other transition metal ions such as Zn^2+^, Cd^2+^, Ni^2+^, Co^2+^ and Pd^2+^, but the response to the intensity change is different. Fixing 334 nm as the excitation wavelength, the fluorescence intensity changes of 385 nm were shown in Figure 9. The response order is Cu^2+^ > Zn^2+^ > Cd^2+^ > Ni^2+^ > Co^2+^, and no response was found with Pd addition at room temperature. When EDTA solution was slowly added into the above dispersing system, the fluorescence intensity at 385 nm was gradually weakened and restored to the state before metal ion addition. These results indicated that DAPD modified magnetic nanoparticles in this work has reversible fluorescence response ability to transition metal ions. The static adsorption capacities of DAPD-SMP were found to be 45 and 32 mg·g^−1^ for Cu^2+^ and Zn^2+^, respectively.

## 3. Experimental

### 3.1. Chemicals and Reagents

Ferric chloride hexahydrate (FeCl_3_·6H_2_O), ferrous sulfate heptahydrate (FeSO_4_·7H_2_O), sodium hydroxide, ammonia solution, anhydrous ethanol, tetraethyl orthosilicate (TEOS) and toluene were purchased from Beijing Chemical Reagent Co., Ltd. (Beijing, China). 3-Chloropropyltrimethoxysilane (CPS) was obtained from Chemical Engineering Corporation of Ocean University of China (Qingdao, China). 2,6-Diaminopyridine (DAPD) was purchased from Shanghai Aladdin bio-chem Technologies Inc (Shanghai, China). All the chemicals were of reagent grade and used without further purification. Distilled water was used throughout the experiment. Standard stock solutions of individual metal ions were prepared by dissolving spectral pure grade nitrate salts (the First Reagent Factory, Shanghai, China) in 1.0% (*v*/*v*) HNO_3_ and further diluted prior to use.

### 3.2. Fabrication of DAPD Modified Magnetic Nanoparticles

#### 3.2.1. Silica-Coated Fe_3_O_4_ Functionalized by –Cl Groups

Fe_3_O_4_ nanoparticles and silica-coated Fe_3_O_4_ magnetic nanoparticles were produced by the conventional co-precipitation and sol–gel method from [24]. The composites prepared (abbreviated SMN) were ultrasonically dispersed into dry toluene. One milliliter of CPS in 5 mL toluene was added to the solution with stirring for 12 h at room temperature. The resulting material was collected and washed with dry toluene and ethanol several times, and finally dried under vacuum.

#### 3.2.2. DAPD Modified Silica-Coated Fe_3_O_4_ Nanoparticles

After washing with ethanol for the last time above, the particles were redispersed in 100 mL ethanol. Then 2.0 g DAPD was added and the mixture was refluxed for 8 h. The particles were separated with the help of the external magnet and washed with ethanol and water several times and dried under vacuum. The SMN functionalized by DAPD were thus obtained and named DAPD-SMN. The schematic diagram of the whole synthetic procedure is given in Figure 1.

### 3.3. Characterization

The morphologies and sizes of the prepared samples were characterized by a JEM-3010 transmission electron microscope (JEOL, Tokyo, Japan). Fourier transform infrared (FT-IR) spectra (4000–400 cm^−1^) in KBr were recorded using Nicolet Nexus 670 FT-IR spectrometer (Nicolet Instrument Company, Madison, WI, USA). Hysteresis loops of these core-shell nanoparticles were recorded by a vibration sample magnetometer (Quantum Design, San Diego, CA, USA). The structure of synthesized products was determined by an X-ray diffractometer using Cu Kα radiation (λ = 1.5406 Å) (Bruker Company, Karlsruhe, Germany). Photoluminescence spectra of all samples were measured at room temperature by a F-4500 fluorescence spectrophotometer (Hitachi Limited, Tokyo, Japan) equipped with a xenon lamp as the excitation light source.

## 4. Conclusions

In this study, the fluorescent property of 2,6-diaminopyridine and magnetic property of iron-oxide nanoparticles were combined through the silica-shell and surface-modification technique. The material is well dispersed and super paramagnetic with a particle size of about 10–12 nm. This nanomaterial possessed magnetic and luminescent properties simultaneously. Furthermore, the material has reversible fluorescence response ability to transition metal ions such as Cu^2+^, Zn^2+^, Cd^2+^ and Ni^2+^. The emission spectrum produced a red shift of 21 nm after transition metal ion addition. The fluorescence intensity gradually increased with an increase in metal ions concentration in certain ranges, which will make the multi-functional nanoparticles more useful in heavy metal ions analysis, detection and monitoring applications.
